# Overview of Bee Pollination and Its Economic Value for Crop Production

**DOI:** 10.3390/insects12080688

**Published:** 2021-07-31

**Authors:** Shaden A. M. Khalifa, Esraa H. Elshafiey, Aya A. Shetaia, Aida A. Abd El-Wahed, Ahmed F. Algethami, Syed G. Musharraf, Mohamed F. AlAjmi, Chao Zhao, Saad H. D. Masry, Mohamed M. Abdel-Daim, Mohammed F. Halabi, Guoyin Kai, Yahya Al Naggar, Mokhtar Bishr, Mohamed A. M. Diab, Hesham R. El-Seedi

**Affiliations:** 1 Department of Molecular Biosciences, The Wenner-Gren Institute, Stockholm University, S-10691 Stockholm, Sweden; 2 Department of Chemistry, Faculty of Science, Menoufia University, Shebin El-Kom 32512, Egypt; esraaelshafiey8@gmail.com (E.H.E.); aya.shetaia@gmail.com (A.A.S.); 3 Agricultural Research Centre, Department of Bee Research, Plant Protection Research Institute, Giza 12627, Egypt; aidaabd.elwahed@arc.sci.eg; 4 Alnahalaljwal Foundation Saudi Arabia, P.O. Box 617, Al Jumum 21926, Makkah, Saudi Arabia; ahmed@alnahalaljwal.com.sa; 5 International Center for Chemical and Biological Sciences, H.E.J. Research Institute of Chemistry, University of Karachi, Karachi 75270, Pakistan; musharraf@iccs.edu; 6 Department of Pharmacognosy, College of Pharmacy, King Saud University, Riyadh 11451, Saudi Arabia; malajmii@ksu.edu.sa; 7 College of Food Science, Fujian Agriculture and Forestry University, Fuzhou 350002, China; zhchao@live.cn; 8 Department of Plant Protection and Biomolecular Diagnosis, Arid Lands Cultivation Research Institute (ALCRI), City of Scientific Research and Technological Applications, New Borg El-Arab City P.O. Box 21934, Egypt; saad.masry@adafsa.gov.ae; 9 Abu Dhabi Agriculture and Food Safety Authority (ADAFSA), Al Ain 52150, United Arab Emirates; 10 Pharmacology Department, Faculty of Veterinary Medicine, Suez Canal University, Ismailia 41522, Egypt; abdeldaim.m@vet.suez.edu.eg; 11 Al-Rayan Research and Innovation Center, Al-Rayan Colleges, Medina 42541, Saudi Arabia; m.halabi@amc.edu.sa; 12 Laboratory of Medicinal Plant Biotechnology, College of Pharmacy, Zhejiang Chinese Medical University, Hangzhou 310053, China; kaiguoyin@zcmu.edu.cn; 13 General Zoology, Institute for Biology, Martin Luther University Halle-Wittenberg, Hoher Weg 8, 06120 Halle, Germany; yehia.elnagar@science.tanta.edu.eg; 14 Zoology Department, Faculty of Science, Tanta University, Tanta 31527, Egypt; 15 Arab Company for Pharmaceuticals and Medicinal Plants, (Mepaco-Medifood), El-Sharqiya 11361, Egypt; m-bishr@mepaco-pharma.net; 16 EWG Company, Menoufia, Shebin El-Kom 32512, Egypt; Mmmdiab82@gmail.com; 17 Pharmacognosy Group, Biomedical Centre, Department of Pharmaceutical Biosciences, Uppsala University, P.O. Box 591, SE 75124 Uppsala, Sweden; 18 International Research Center for Food Nutrition and Safety, Jiangsu University, Zhenjiang 212013, China

**Keywords:** bees pollination, economic, crop production, bee visitation, challenges, impact

## Abstract

**Simple Summary:**

There is a rising demand for food security in the face of threats posed by a growing human population. Bees as an insect play a crucial role in crop pollination alongside other animal pollinators such as bats, birds, beetles, moths, hoverflies, wasps, thrips, and butterflies and other vectors such as wind and water. Bees contribute to the global food supply via pollinating a wide range of crops, including fruits, vegetables, oilseeds, legumes, etc. The economic benefit of bees to food production per year was reported including the cash crops, i.e., coffee, cocoa, almond and soybean, compared to self-pollination. Bee pollination improves the quality and quantity of fruits, nuts, and oils. Bee colonies are faced with many challenges that influence their growth, reproduction, and sustainability, particularly climate change, pesticides, land use, and management strength, so it is important to highlight these factors for the sake of gainful pollination.

**Abstract:**

Pollination plays a significant role in the agriculture sector and serves as a basic pillar for crop production. Plants depend on vectors to move pollen, which can include water, wind, and animal pollinators like bats, moths, hoverflies, birds, bees, butterflies, wasps, thrips, and beetles. Cultivated plants are typically pollinated by animals. Animal-based pollination contributes to 30% of global food production, and bee-pollinated crops contribute to approximately one-third of the total human dietary supply. Bees are considered significant pollinators due to their effectiveness and wide availability. Bee pollination provides excellent value to crop quality and quantity, improving global economic and dietary outcomes. This review highlights the role played by bee pollination, which influences the economy, and enlists the different types of bees and other insects associated with pollination.

## 1. Introduction

Pollination plays a vital role in maintaining the natural balance of ecosystems and is the cornerstone of crop production, providing a link between agriculture and the cycle of life. Consequently, pollination has a role in the economic sector owing to the improvement of quality and quantity [1,2,3].

Pollination is defined as the process by which pollen moves from the male anthers to the female stigmata, either within the same flower (self-pollination) or between plants (cross-pollination) [4,5]. Pollinators are the key players of the crop yield process since plants completely rely on vectors to transfer their pollen in cross-pollination. For instance, incorporating both wild and managed bee species in a region could enhance cross-pollination [6]. Possible other vectors include water and wind, and animal pollinators involve bats, birds, butterflies, hoverflies, wasps, thrips, diptera, and other animals [6,7,8,9].

Animal pollinators contribute to the production of 87 global crops, including cocoa (*Theobroma cacao*), kiwi *(Actinidia deliciosa* var. deliciosa), passion fruit (*Passiflora edulis*), and watermelon (*Citrullus lanatus*) from 200 countries. Thirty percent of these crops participate in global economic food production. Global pollination’s economic value averaged EUR 153 billion, which is worth 9.5% of the world’s agricultural production of human food in 2005. The leading categories of insect-pollinated crops are vegetables and fruit, making around EUR 50 billion each, followed by edible petroleum crops, stimulants, nuts and spices. The one ton of crop production that is not dependent on insect pollination is valued at about EUR 151, compared to an average of EUR 761 for crops dependent on pollinators [10]. Pollination by insects is a key element in the production of a large number of agricultural products worldwide, including aromatic and medicinal plants such as black cumin (*Nigella sativa* linn), cumin (*Cuminum cyminum* linn) [11], anise (*Pimpinella anisum* linn) [12], sunflower (*Helianthus* spp.) [13], and coriander (*Coriandrum sativum* linn) [14]. Each season, honey bees, local bees, and flies pollinate 48 crops of the world’s most valuable commodities, contributing significantly to the global economy. [15]. For instance, in the USA alone, pollination results in USD 16 billion annually with USD 12 billion attributable solely to the accessibility of honey bees [16,17].

The Western honey bee (*Apis mellifera* L.) is the main species responsible for bee pollination worldwide and meets, for instance, 34% of pollination service demands in the United Kingdom [2,18]. Although several other bee species also contribute to pollination, researchers have focused on only a limited number of these to date, particularly the bumble bee (*Bombus* spp.) [19,20]. In comparison to wild bees alone, Greenleaf and Kremen observed that interactions between wild bees and honey bees doubled pollination rates and enhanced the prevalence of hybrid sunflowers by five-fold [21].

This review aims to highlight the role of the bee in plant pollination and its impact on the economy. The factors influencing bee visitation of flowers and plants, in addition to a comparison of bees and other insect pollinators, are reported.

## 2. Effect of Bee Pollination on the Economy

There is an ever-increasing demand for food security in the face of challenges such as climate change, land-use changes, habitat transformation, and the expanding human population. Proper pollination can improve the quantity and quality of fruits, nuts, oils, and other crops produced [22]. According to market prices, pollination by animals improves the global crop output by an additional USD 235–577 billion annually, with the greatest economic benefits having been seen in the Mediterranean, Southern and Eastern Asia, and Europe [23]. However, greater production also leads to an increased demand for pollination services [24]. Around the world, 5–8% of crop production would be lost without animal pollination [25], and pollination also provides many services to ecosystems, such as enhancing biodiversity and increasing food production without threatening the environment [26].

Bees are the main pollinators of plants. According to Gallai et al., insect pollination provided EUR 153 billion, representing 9.5% of the total economic value of agricultural production used directly for human food [10]. Consequently, countries that grow cash crops such as coffee (*Coffea* spp.), cocoa, almond (*Prunus dulcis (*(Mill.)), and soybeans (*Glycine max* L.) have a much greater reliance on pollination in agriculture at a large scale [27,28,29]. Scientists have used several methods to estimate the annual benefit of certain ecological costs incurred by native insects in the USA, which have been shown to amount to more than USD 57 billion, USD 3.07 billion of which is a result of bee pollination [30]. The pollination services of non-apis pollinators were valued at USD 3.44 billion, but honey bees contributed approximately USD 11.68 billion by 2009 in USA [16]. Honey bees are responsible for pollinating over 100 commercial crops in North America [3]. Both honey bees and wild bees are also economically important for sunflower seed production, which is an uprising industry estimated at approximately USD 10.4 million annually [21].

Bee pollination also increases the yield of crops cultivated in farmland. For instance, in sub-Saharan Africa, which is considered the main producer of cotton [29,31], bee pollination increases the cotton yield to 62% compared with an estimated 37% without bee pollination [32]. In addition, economic returns from bee pollination have been recorded in smallholder farming systems in Kakamega (western Kenya), where several crops benefit from pollination, including green gram (*Vigna radiata*), beans, cowpea (*Vigna* *unguiculata* L. Walp), sunflower, tomato (*Solanum lycopersicum* linn), bambara groundnut (*Voandzeia subterranean* L.), passion fruit, and capsicum, with pollination dramatically improving the production rate and being responsible for almost 40% of the annual crop production [33].

The estimated annual value of pollination services rendered by bees in Brazil’s protected areas in 2016 was approximately USD 564,000 in the north (Serra da Bocaina, Pará) and USD 246,000 in the southeastern region (Mata do Jambreiro) [34]. Of the 36 crops produced in the state of Pará, 20 (55%) are dependent on animal pollinators, and the overall value of pollination services was USD 983.2 million in 2016, equating to 33% of the total value of crop production (USD 2.95 billion). Four groups represented for 96% of Pará’s pollination service value including; cocoa (USD 187.6 million), Acaí palm (USD 635.6 million), watermelon (USD 26.1 million), and soybean (USD 98.4 million) [35]. In the USA, wild bees and honey bees have produced comparable quantities of pollination for most crops, including in agriculturally-intensive areas. The annual production value of wild pollinators for seven crops is over USD 1.5 billion. The value of wild pollinators is estimated to be the largest in apples, with a value of USD 1.06 billion while the approximate values of watermelon (USD 146 million), blueberry (USD 50 million), sweet cherry (USD 145 million), art cherry (USD 32 million), and pumpkin (USD 101 million) are evidentially high. The economic value of honey bees on yield across these crops is about USD 6.4 billion [36].

## 3. Role of Bee Pollination in Crop Production (Quality and Quantity)

The number of visits and the aggregate effects of various bee species influence not only the quantity of crops produced but also their quality, which is important mainly from an economic perspective [37]. Plant pollination by more than one bee species, including honey bees, carpenter bees, stingless bees, bumble bees, long-tongued bee, feral bees, social bees, and solitary bees, results in a better pollination/vegetation process, as shown in Table 1.

### 3.1. Honey Bees

Western honey bee have been widely used as pollinators since the application of pollination services began, and are the primary managed species worldwide for both honey production and crop pollination [38]. Indeed, the Western honey bee ranks as the single most popular species of pollinator for crops globally [39], and is the most effective crop visitor worldwide, contributing approximately 13% of floral visits to 5% of plant species across all plant networks [40]. However, there are at least eight other honey bee species in the genus *Apis*, such as *A. florea* Fabr., *A. cerana* Fabr., *A. andreniformis*, and *A. dorsata* Fabr [41]. In 2009, it was estimated that honey bees contributed USD 11.68 billion to agriculture in the USA [16].

Honey bees are considered significant pollinators due to their effectiveness and wide availability [16]. The mutualistic relationship between plants and honey bees results from the exchange of nectar and pollen. Bees play a vital role in the pollination of plants [40], and plants secrete a rich liquid sugar similar to nectar from their glands to attract pollinators to their flowers so that the pollen can adhere to bee-collected pollen grains [42]. Researchers have found that honey bees (*A. mellifera* L.) appear to prefer crops rich with nectar and pollen in order to store large quantities of food, thus sustaining the colony growth and improving foraging performance [43,44].

Many countries have used honey bees and achieved great results in terms of the quality and quantity of crops, as shown in Table 1. In the USA, the pollination activity of honey bees is well recognized for three species of crops: cucumber (*Cucumis sativus* Linn), for which there has been a 10% increase in yield and the number of colonies has increased from 40,000 to 45,000; cranberry (*Vaccinium oxycoccos* Linn), which experienced an increase in yield from 3.7 million in 1989 to 5.4 million in 1998 [45], and pear (*Pyrus communis* Linn), which exhibited a 7% increase in fruit size and a net income increase of $400 per hectare [46]. In India, the use of honey bees as pollinators improved the fruit quality of guava (*Psidium guajava* Linn), as well as the fruit length and girth of coconut (*Cocos nucifera* Linn) and citrus (*Citrus* spp.) compared with the controls [47,48]. In Egypt, honey bees have significantly improved the seed set percentage and seed yield in onion (*Allium cepa* Linn) crops compared with other insects [49]. Furthermore, in Burkina Faso, the production of sesame (*Sesamum indicum* Linn) seeds tripled after using honey bees as pollinators [32].

The pollination of oilseed rape (*Brassica napus* Linn), buckwheat (*Fagopyrum esculentum* Moench), and strawberry (*Fragaria* × *ananassa* (Duchesne ex Weston) Duchesne ex Rozier) have clearly been dominated by honey bees, which have improved their quality and yield [50]. Similarly, black cumin flowers are attractive to a range of pollinators, such as Hemiptera (true bugs), Coleoptera (beetles), Diptera (flies), and Hymenoptera (bees) [51]. However, honey bees are the most abundant pollinators affecting its productivity and quality [11], with their pollination activity increasing the number of seeds and affecting the total yield, which has led to the recommendation that beekeepers place bee colonies near black cumin fields for better pollination [52].

The yield of anise also significantly relies on pollinator activity. One study showed that honey bees exhibited a daily peak in anise pollination activity between 12 noon and 2 p.m., and increased the yield above levels seen with insect exclusion, though levels were below those obtained with open pollination [12]. Honey bees and six species of Andrenidae are the main pollinators of coriander, with 63% of honey bee visits and 100% of the visits by three species of Andrenidae resulting in pollinating activity [53].

For the apple (*Malus domestica* Borkh), increased flower visitation rates by high-quality honey bee colonies increased fruit set by 15%, as well as the fruit sugar content and seed set compared with visits by conventional colonies, resulting in the farmer’s profits increasing by 70%. Pollination by high-quality colonies also increased fruit weight by approximately 20% [54]. In the fruit of cape gooseberry (*Physalis peruviana* Linn), western honey bees’ pollination improved the equatorial diameter by a mean of 13.3%, fruit mass by 30.3%, seed variety by 7%, and seed mass by 8.4% compared with self-pollination [55], while the use of honey bees for almond pollination increased fruit set by 60% compared with bee-remote trees, which translated into a 20% increase in yield [29]. Observations of blueberry (*Vaccinium corymbosum* Linn) pollination in the presence of wild bees (Bombus spp., Halictids bees, Andrenids bees, and *Xylocopa virginica*) and controlled honey bees in small isolated and large fields in Michigan, USA, showed that wild bees were the primary pollinators in the small fields, accounting for 58% of flower visits, whereas honey bees were the main pollinators in the large fields, accounting for 97% of visits. Furthermore, it was found that flowers in the large fields were visited by four times as many bees as flowers in the small fields. The weight of the fruit was affected by the level of bee pollination and the abundance of bees, and the weight of berries was twice as high in the large fields compared with the small fields [56].

### 3.2. Bumble Bees

Bumble bees (Apidae: Bombini) are vital pollinators for agricultural and wild plants worldwide, and their pollination supports food security [57]. Five species of bumble bees are generally used for pollination of commercial crops: *Bombus terrestris* Linn (in Europe, North Africa, Asia, and Australasia), *B. occidentalis* Greene (in western North America), *B. ignitus* and *B. lucorum* Linn (in East Asia), and *B. impatiens* Cresson (in North America) [58].

The strong adaptation to different climates and habitats of bumble bees explains their ability to continue foraging even in high and low temperatures [59]. Bumble bees have contributed to the crop pollination via increasing the yield and enhancing the quality of fruits [60]. Indeed, fruit growers gain many benefits from pollination by bumble bees, which are good pollinators of several crops, such as kiwifruit (*Actinidia Deliciosa*) [61], sweet pepper (*Capsicum annum* Linn) [62,63], and red clover (*Trifolium pretense* Linn) (Table 1) [19].

Bumble bees are important pollinators of a diverse range of crops, including buzz-pollinated crops, such as blueberry and tomato, as well as both large-flower and small-flower crops, giving them the potential to be sufficient pollinators in open fields and greenhouses [64,65]. It has also been shown that buzz pollination by *Bombus haemorrhoidalis* Smith in India leads to bigger, longer, heavier, and healthier fruits, especially in kiwi fruit [61].

Pollination by bumble bees enhances the quality and quantity of tomato fruit, including the number of fruit per cluster, the number of fruit per plant, fruit length, fruit freshness, fruit breadth, and fruit yield (Table 1) [65]. In addition, pollination of sweet pepper by bumble bees results in a larger number of pollen grains and a higher level of seed set on the fruit than self-pollination, such that flowers visited by bumble bees produce larger and heavier fruit than non-visited flowers [63]. Finally, bumble bees have provided maximum pollination services to hybrid leek (*Allium porrum* Linn), resulting in a 25% increase in plant quality, which has influenced the plant quality and crop price value by an estimated USD 18,007 and USD 17,174 hectare, respectively [60]. In some cases, wild pollinators give better pollination than honey bees, as seen in apple crops pollinated by bumble bees, because all wild bee species are able to hold and deposit more apple pollen than honey bees [66].

### 3.3. Stingless Bees

Stingless bees (Apidae: Meliponini) are common floral visitors in tropical and subtropical areas around the world. They exhibit greater dietary diversity and intensity in their foraging behavior than honey bees and so are likely to influence the future development of pollination solutions that are best suited to the needs of particular crops and habitats [67].

Stingless bees are a large, diverse group of eusocial bees, making them good candidate pollinators. They vary widely in their body size, being described as small- to medium-sized, and have vestigial stings [67]. Some species tend to be large and smooth, with long hairs that help to bring pollen and other products to the colony [68]. The physiology of stingless bees is suited to flower pollination [69] because they have suitable structures for collecting pollen, nectar, and an absence of stinging behavior, making them easier to handle than the majority of honey bees. Some stingless bees, such as those in the genus Melipona, exhibit vibration behavior to extract the pollen, which is needed in crops with poricidal anthers, such as tomato and pepper [70].

The neotropical stingless bee *Melipona quadrifasciata* Lepeletier is used to pollinate greenhouse tomatoes, and has improved the production of fruit with lower levels of mechanical injury [71]. Stingless bees also play a prominent role in the pollination of greenhouse cucumber crops, improving both the fruit weight and yield [72]. The pollination of cucumbers by the stingless bee *Heterotrigona itama* and manual cross-pollination improved crop quantity and fruit quality, allowing heavier, longer, and wider fruit to be produced [73]. Similarly, the pollination of rockmelon (*Cucumis melo* var. reticulatus) by stingless bees and manual cross-pollination had a positive effect on fruit set and the number of seeds per fruit compared with self-pollination [74], and the pollination of strawberries in greenhouses by stingless bees increased the quality and commercial value of the fruit compared with a control group [75]. Furthermore, the pollination of eggplant (*Solanum melongena* Linn) by *Melipona fasciculata* Smith in greenhouses increased fruit set by 29.5% and increased fruit quality (measured as fruit weight) compared with self-pollination [76].

### 3.4. Carpenter Bees

Large carpenter bees are a group of bees that occur in tropical and subtropical areas and belong to the genus Xylocopa in the tribe Xylocopini (Apidae: Xylocopinae) [77]. Compared with other non-*Apis* bees, carpenter bees have numerous advantages in crop pollination, as they feed on a broad range of plant species during their long activity seasons. They also have the ability to buzz-pollinate flowers, making them even more diverse crop pollinators [78]. However, there is a great need for a sufficient breeding program to be developed that involves the selection of genotypes, controlled mating, and nest foundation [79].

Carpenter bees are known for their ability to make their nests in tunnels in hard wood, logs, stumps, or the dead branches of trees [80]. In India, carpenter bees are active throughout the year and forage on a variety of flowers during the day and sometimes even work through moonlit nights. It has been noticed that flowers visited by carpenter bees produce nectar that is odoriferous, so it is possible that these bees use this odor as a cue to visit the correct flowers [81].

The use of carpenter bees for pollination services is necessary to guarantee adequate pollination for several crops, including passion fruit (*Passiflora edulis* f. flavicarpa), cucurbits, and other vegetables and fruits, as observed in the Philippines, Brazil, USA, and Malaysia [82,83]. Yellow passion fruit is satisfactorily pollinated when the flowers are only visited by native bees, especially carpenter bees [84]. Furthermore, when native carpenter bees (*Xylocopa (Lestis*)) were used as an alternative to bumble bees for tomato pollination in a greenhouse, the females visited and buzz-pollinated the flowers and the resulting fruit were heavier and contained more seeds than those that were not pollinated by these bees [85]. The carpenter bee *Xylocopa pubescens* Spinola is also used to pollinate greenhouse-grown honeydew melons (*Cucumis melo* Inodorus Group), as it was noticed that while this species had shorter visit durations per flower than the honey bee, pollination by both bees resulted in a similar fruit mass and seed numbers, and *X. pubescens* pollination increased fruit set three-fold compared with honey bee pollination [86].

### 3.5. Solitary Bees

Solitary bees comprise the majority of bee species in the world. Solitary bee species account for 85% of all bee species [87].The majority of solitary bees are polylectic (i.e., collect pollen from numerous plant species), while a smaller number are oligolectic (use a narrow range of plants) and very few are monolithic (use only a single plant species). In recent decades, there has been a decline in monolithic and oligolectic species in Britain [88]. Solitary bees play a major role in pollination, and it has been demonstrated that wild bees contribute USD 3251/hectare for their pollination services worldwide, seven out of ten of which are solitary [89]. Solitary bees are more effective pollinators than honey bees for some crops that depend on pollinators for their reproduction, such as apple. Indeed, in the United Kingdom, the economic gains of using solitary bees for apple production were estimated to be € 51.4 million compared to honey bees of € 21.4 million [90].

**Table 1:** The impact of bee pollination on crop quality and productivity in various countries.

Crop (Species)	Bee Pollinator	Impact on Crop Yield	Country	Reference
**Fruits**				
Apple (*Malus* *domestica* L.)	Honey bees (*Apis mellifera* L.)	Enhancing fruit production with high yield and quality (fruit size and number of seeds).	Pakistan	[91]
	Wild bees and honeybees (*A. mellifera*)	Seed number increased with bee abundance which consequently increased fruit quality.	China	[92]
	Stingless bees (*Melipona quadrifasciata anthidioides* Lepeletier) Africanised honeybee (*A. mellifera*)	Both stingless bees (12 hives/hectare) and Africanized honeybees (7 hives/ hectare) provided higher seed and fruit production than supplementation with honeybees alone.	Brazil	[93]
	Honey bee (*A. mellifera*)	Increased fruit set by 15%, seed set and content of fruit sugar, and farmer’s profits by 70%.	Argentina	[54]
	Bumble bees (*B. impatiens*) and honey bee (*A. mellifera*)	The quantity and quality of fruits produced from pollination from both species were equivalent.	Canada	[66]
	Wild bees	Fruit set increased	USA	[94]
Coconut (*Cocos nucifera* L.)	Honey bees (*A. mellifera*)	Increased fruit set	Mexico	[48]
	Honey bees (*A. mellifera*)	Effective pollinators compared to wasp	Jamaica	[95]
Watermelon (*Citrullus lanatus* Thunb.)	Honey bees *(A. mellifera*)	Fruit set, fruit numbers and weights per plot increased linearly as number of honey bees visits increased.	USA	[96]
Tart cherry (*Prunus cerasus* L.)	*Osmia lignaria* solitary bee	Cherry weight increased by 2.8% compared to the control.	Utah	[97]
Cape gooseberry (*Physalis peruviana* L.)	Honey bees (*A. mellifera*)	Improvement of fruit mass by 30.3%, equatorial diameter by 13.3%, seed variety by 7%, and seed mass by 8.4%.	Colombia	[55]
Sweet cherry (*Prunus avium L*.)	Wild bees and honey bees	Fruit set was enhanced compared to open pollination.	Germany	[98]
Almond (*Prunus dulcis* (Mill.) D.A.Webb)	Honey bees (*A. mellifera*)	Increased fruit set by 60% and kernel yield by 20% compared to self-pollination.	USA	[29]
	Solitary Bee (*O. cornuta*)	Increased fruit production was parallel with increased visits by *O. cornuta.*	Spain	[99]
Avocado (*Persea americana* Mill.)	Honey bees (*A. mellifera*)	High pollination efficiency for fruit set, increased the production, and improved the weight of the fruit.	In Central America	[100]
Passion fruit (*Passiflora edulis* Sims. f. flavicarpa Deg)	Honey bees *(A. mellifera*), and carpenter bees (*Xylocopa* spp.)	The diversity of bee species affected the fruit set and fruit quality and led to a higher reproductive efficiency.	Australia and Philippins	[82,83]
	Native Brazilian bees (*Xylocopa* spp.)	Production costs lowered by 58%. Average production was 7000 kg/hectare/year.	Brazil	[101]
Citrus (*Citrus sinensis* L.)	Honey bees (*A. mellifera*)	Lead to heavier fruit with less acid content and fewer seeds per bud.	Brazil	[102]
Mango (*Mangifera indica* L.)	Honey bees (*A. cerana*)	Fruit setting was 42.29% compared to open pollination 33.36%.	India	[103]
Guava (*Psidium guajava* L.)	Honey bees (*A. mellifera*)	Increased fruit set; improved the quality of fruit length and girth.	India	[47]
Strawberry (*Fragaria × ananassa DUCH)*	*Osmia bicornis* L.	Increased commercial value by 54.3% compared with self-pollination and by 38.6% compared with wind pollination. Number of fertilized achenes increased, and improved post-harvest quality occurred (more intensive red colour and lower sugar acid ratios).	Germany	[104]
	Bees	Quantity and quality improved. Yield increased 20%.	Germany	[50]
	European orchard bee (*Osmia cornuta* Latr)	Fruit weight was higher than the control treatment.	Germany	[105]
Kiwifruit (*Actinidia Deliciosa*)	Honey bees (*A. mellifera*)	Increased fruit set and yield.	Australia	[106]
	Bumble bee *(Bombus haemorrhoidalis* Smith)	Higher fruit breadth, longer fruits, heavier fruits, higher healthy fruits, and higher fruit set.	India	[61]
Pear (*Pyrus communis* L.)	Honey bees (*A. mellifera*)	Fruit size increased by 7% and lead to USD 400 per hectare net increase in income.	USA	[46]
Cranberries (*Vaccinium oxycoccos* L.)	Honey bees (*A. mellifera*)	Production increased from 3.7 million in 1989 to 5.4 million in 1998.	USA	[45]
**Vegetables**				
Cucumbers (*Cucumis sativus* L.)	Honey bees (*A. mellifera*)	10% increase in production.	USA	[45]
	Stingless bee (*Heterotrigona itama*)	Lead to larger, heavier, and longer cucumbers.	Terengganu	[73]
Sweet pepper (*Capsicum annuum* L.)	Bumble bee (*Bombus impatiens* Cr.)	Increased fruit weight, width, and volume. Increased seed weight and reduced harvesting time.	Canada	[62]
	Bumble bees (*Bombus terrestris* L.)	Increased yields, fruit weight, and quality of seed, and fruits under unheated greenhouse conditions. Seed set was 49.8% compared to 27.5% of the control (self-pollination) treatment.	Spain	[63]
Tomatoes *(Solanum lycopersicum* L.)	Bumble bee (*Anthophora urbana* Cresson and *Bombus vosnesenskii* Radoszkowski)	Lead to higher yield and improved the quality of fruits.	USA	[107]
	Bees (*Exomalopsis analis Spinola*, *Centris tarsata Smith*, *Bombus morio Swederus*, *Eulaema nigrita Lepeletier* and *Epicharis* sp.)	Increased fruit production and quality.	Brazil	[108]
**Aromatic and medicinal plants**				
Anise (*Pimpinella anisum* L.)	Honey bees (*A. mellifera*)	Increasing seed yield/feddan to 781.55 kg compared to 300.24 Kg for control group (insect exclusion).	Egypt	[12]
Black Seed (*Nigella sativa* L.)	Honeybee (*A. mellifera*)	Increased yield and seed setting but no effect on the weight of the seed produced.	Pakistan	[109]
Cumin (*Cuminum cyminum* L.)	*Apis florea* F., *A. mellifera* and *A. dorsata*	Enhanced yield by 40.03% compared to 41.37% for open pollination.	India	[11]
Sunflowers (*Helianthus annuus* L.)	Wild bees and honey bees (*A. mellifera*)	Interactions between wild and honey bees increased the efficiency of pollination up to 5-fold compared to honey bees only.	USA	[21]
	Africanized honey bees (*A. mellifera*)	The average yield of seeds was 43% higher compared to the control.	Brazil	[13]
	Honey bees (*Apis mellifera* L.)	Played a significant role in pollination compared to moths and wind.	Central Darling Downs	[110]
Coriander (*Coriandrum sativum* Linnaeus.)	*Apis cerana* Fabricius	The seed set was significantly higher by 69.51% compared to 54.89% in the control group. The yield was 14.57 q/hectare vs 11.66 q/hectare in the control group.	India	[14]
**Other plants**				
Cotton *Gossypium hirsutum* L.)	Honey bees (*A. mellifera*)	Increased production by more than 12% for fiber weight and over 17% for seed number.	Brazil	[111]
	Honeybees and wild bees	Significantly increased yield quantity and quality by an average of 62%. The average yield was 953.91 kg/hectare.	West Africa	[32]
Pumpkins (*Cucurbita maxima* L.)	Honey bees (*A. mellifera*)	Fruit set, fruit size, weight, and number of seeds increased linearly with the number of visits.	Brazil	[112]
Soyabean *(Glycine max* L.)	Honey bees (*A. mellifera*)	Yield increase was associated with an increase of the seed number.	Argentina	[113]
	Honey bees (*A. mellifera*)	Increased yield by 18.09%.	Brazil	[114]
Sesame (*Sesamum indicum* L.)	Honeybees (*A. mellifera*) and wild bees	The mean yield of seed was 202.20 kg/hectare. The exclusion of pollinators caused an average yield gap of 59%.	West Africa	[32]
*C. canephora* L	*Apis dorsata* F.	Bees increased fruit production of coffee by 50% more than wind.	South India	[115]
Cowpea (*Vigna unguiculata* L. Walp)	Honey bees and bumble bees	NR	Nigeria	[116]
Red clover seed (*Trifolium pratense* L.) legume	Bumble bee (*B. vosnesenskii)*	High yield and most production of seeds.	USA	[19]
Pineland golden trumpet *(Angadenia berteroi* (A.DC.) Miers)	Long-tongued bee (*Megachile georgica* Cresson and *Melissodes communis communis*)	NR	USA	[117]
Mustard (*Brassica juncea* L.)	Honey bees (*A. mellifera*)	Increased fruit set, viability of seed, seed yield, and oil nutrient contents in the seed.	India	[118]
	Honey bees (*A. cerana*)	Increased siliqua/panicle by 20.8%, seeds/silique by 9.4%, and seed yield by 17.1% compared to open pollination.	India	[119]
Green grams (*Vigna radiate* L.) and Bambara groundnut (*Voandzeia subterranean* L.)	Feral bees	Enhanced yield and improved the quality of crops.	Kenya	[33]
Coffee (*Coffea arabica* L.)	Solitary bees and social bees	Significantly increased fruit set.	Indonesia	[120]
Acai palm (*Euterpe oleracea* Martius)	Stingless bee (*Scaptotrigona aff. postica*)	Increased the production reach to 2.5 times. The increase was evident as per the number of fruits per bunch and fruit size.	Brazil	[121]
Oilseed rape (*Brassica napus* L.)	Solitary mason bee (*Osmia rufa* L.)	Increased fruit set, yield, and the number of seeds per pod by bee density.	Germany	[122]
	Honey bees (*A. mellifera*)	Increased oil and decreasing chlorophyll content.	Sweden	[50]
	Honey bees (*A. mellifera*), and wild bees (*Lasioglossum* spp.).	Average yield was increased up to 37.5%.	France	[123]

NR: Not reported.

## 4. Bee Visitation

**Figure 1:**
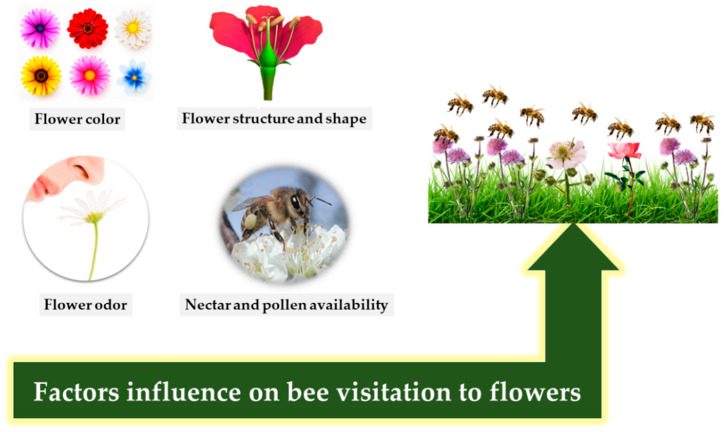
Factors that influence bee visitation.

Pollination is carried out by bees and other insects for a variety of plants. Because pollination is so important to plants, they adapt to be more appealing to pollinators [124,125]. Plants possess several means to attract bees, including flower color [126,127], flower motion as in the case of bumble bees [128], the type of plant cells (e.g., conical epidermal cells) as in bumble bees [129], visual and olfactory cues as in honey bees and apple pollination [130] and the production of nectar and pollen grains [131,132], as shown in Figure 1. Thus, plants play an important role in influencing the visitation rate of their pollinators [133].

One of the most important properties of plants that attracts bees is the color of the flowers [134,135]. Bees have a trichromatic visual system that is sensitive to green, ultraviolet, white, and blue wavelengths, allowing them to see numerous colors. Bees often visit blue or purple flowers but mostly prefer blue flowers [136,137]. By contrast, bees are less attracted to red flowers, though they will sometimes visit red flowers that reflect ultraviolet light [138].

The motion of flowers is also crucial for enhancing attractiveness to bees, as this is integral to their three-dimensional vision. The relative motion of the flowers increases the number of opposing stimuli on the bee‘s eye. Conical epidermal cells have multiple functions, such as promoting the perception of flower color and reducing the wettability of the petal, which increases the effectiveness of the pollination process. Consequently, bees usually prefer to visit conical-celled *Petunia* flowers, particularly those that are most attractive with motion [139,140]. Indeed, higher numbers of bees occur on the moving portion of a flower bed due to their positive response to optical stimulation [139]. In addition, floral volatile compounds affect the orientation of bees [141], as well as pollen collection and behavior in bumble bees [142]. Flower size has a special function for attracting pollinators [141]; bumble bees (*Bombus diversus* Smith) prefer large floral displays [143].

Finally, the quantity and/or quality of pollen and nectar produced may affect the visitation of flowers by bees. Nectar and pollen are sources of energy, protein, and lipids for bees, and other pollinators [144] and bees are drawn to plants to provide food for their young [145]. Solitary bees search for pollen but rarely nectar [132], whereas honey bees search for flowers with larger amounts of nectar [146]. It has previously been shown that the composition of wild bee populations that visit various plants can best be explained by variations in the flowering areas, height of the flowers, and amount of pollen deposited in the flowers [147]. For example, British bumble bees differentiate between *Mimulus guttatus* plants on the basis of their pollen content and quality, with a significant association between visitation and pollen content [148]. Furthermore, both wild bees and honey bees forage on sunflowers for their nectar sugar, with the number of flower visits increasing significantly with an increasing nectar sugar level and decreasing during the corolla period but seeming to be unaffected by nectar sugar composition. Wild bees make more visits to sunflowers that provide pollen (male-fertile plants) and honey bees favor pollen-free flowers (male-sterile plants) [146]. Cover crops, particularly low-diversity mixes that include buckwheat and *Phacelia* spp., provide a high abundance of flowers throughout the summer, resulting in excessive bee visitation rates, with *Phacelia* spp. being more appealing to honey bees and bumble bees, while sunflowers and local wildflowers are more appealing to solitary bees [149].

Honey bees visit native and cultivated plants at comparable average rates that are independent of floral abundance, therefore increasing their visitation rates for the highly abundant plants, whereas the visitation rate of wild pollinators is higher for cultivated plants than for native plants. For example, knapweed (*Centaurea* spp.) is a widespread and often locally important plant for honey bees, as it is favored and regularly visited for its pollen and nectar [150]. In France, the visitation ratio of large solitary bees, wild beetle pollinators, and bumble bees was negatively impacted by the abundance of honey bees colonies [151].

One behavior that some bees have developed during their visitation is buzz pollination, whereby the bees make vibrations to remove and collect the pollen from the fruit set, fruit mass, and flowers during fertilization [152]. About half of bee species can perform buzz behavior, such as large carpenter bees, minute sweat bees and bumble bees, but they differ in their buzz properties [153,154]. This vibration is the best means for extracting pollen from plant species that have small pores on their anthers [155]. During this behavior, the bee bites the anther of the flower and makes vibrations with its thoracic muscles while in direct contact with the flower, causing the vibrations to be transmitted into the flower [156]. There are several ways of performing this buzzing behavior. For example, the flowers of *Pedicularis* spp. have long anthers and narrow corolla tubes, so bees make their vibrations on these corolla tubes for rapid pollen extraction [157,158]. Therefore, this behavior is related to the functional specificity of flowers, particularly in those flowers in which pollen release requires modification of the stamens. Buzz pollination takes its name from the audible sound made during the vibration and is also often called sonication [157]. It is currently believed that this vibration behavior for pollen collection is not performed by any other animal [70]. However, further research is required to determine whether some flies also use vibration behavior to collect pollen.

Most bees visit flowers during the daylight; only five of nine families of bees search for flowers in dim light [159]. Nocturnal bees have different factors that affects their flower trips, including light intensity and temperature. Temperatures and light levels are lower at night than during the day, and this can affect, in particular, the behavior of nocturnal bees [29].

## 5. Challenges Faced in Bee Pollination

**Figure 2:**
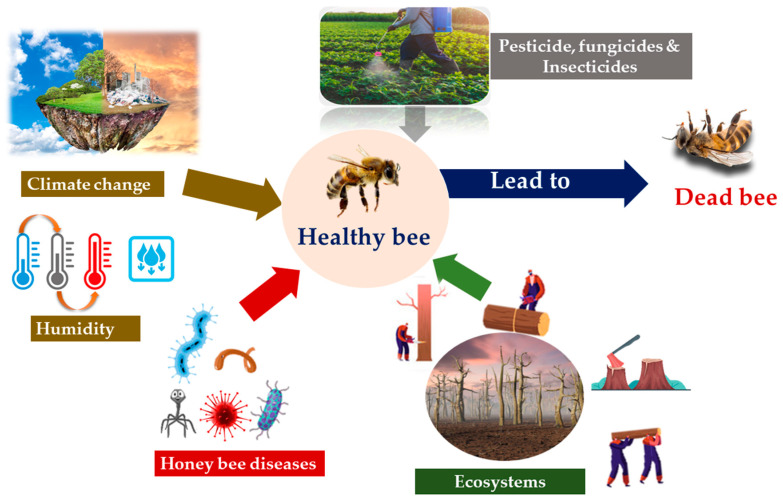
Challenges faced in bee pollination.

Bees are surrounded by several variables that affect their role as pollinators, such as pathogens, nutritional shortages, climate change, and deforestation (Figure 2) [160,161,162,163]. Pathogens such as viruses and bacterial infections have a negative effect on bee health and longevity, threatening pollination services of crops and wild plants [164]. Viral infections affect bee immune systems, causing disease in entire colonies [165]. Colony collapse disorder (CCD) is a phenomenon whereby there are unexplained, rapid losses of adult working bees in managed bee colonies (e.g., honey bee colonies in the USA), resulting in only the queen and a few nursing bees remaining [166]. This problem faces many beekeepers [167]. In the USA, the parasites *Nosema ceranae* and *Nosema apis* also have highly pathogenic effects, causing huge honey bee losses [168]. Conroy et al. [169] found that both nutritional limitation and pathogens have a large effect on bumble bees, with a lack of pollen and low nectar sugar levels leading to reduced pollination and, consequently, a decline in production. In addition to the natural factors affecting bee pollination, the use of pesticides, such as acetamiprid and ergosterol-inhibiting fungicides, threaten pollination services [170]. The residues of pesticides and other synthetic products remain in the nectar and pollen collected by bees, leading to neurotoxicity, immune deficiency, behavioral changes, and chronic ailments [171,172]. The application of neonicotinoid insecticides, which are systemic insecticides that are transferred into the pollen and nectar of many pollinated crops, is one of the main co-factors associated with bee losses [173,174]. Spraying agrochemicals such as fungicides, insecticides, and pesticides cause contamination, toxicity, and declines in the quality and quantity of nutrients in the pollen and nectar, leading to poor colony health and hence threatening the survival of bees [175,176].

Recently, the Environmental Protection Agency (EPA) has been investigating the effect of pesticides on the immune system of bees [177], while the European Food Safety Authority assessment provides information on the chronic toxicity of pesticides on bees [178]. The effect of neem-based insecticide (botanical) and pyrethroid insecticides, deltamethrin and the fungicides thiophanate-methyl and chlorothalonil (synthetic) insecticides on the melon (*Cucumis melo* L.) has been investigated. Both insecticides and pesticides not only reduced the visitation intensity of bees but also lead to lower melon yield [179]. Therefore, good nutrition has a direct effect on the immune function and an indirect effect on energy availability [161].

Many factors also affect the growth, reproduction, and survival of bees, such as high temperatures, and humidity, reducing not only the biodiversity of bees and other pollinators but also agricultural production [160]. Most bees visit flowers during the daylight, but some bee families search for flowers in dim light [159]. Nocturnal bees settle their flower trips by light intensity and temperature [180].

Deforestation can also affect bee populations (Figure 2) [163]. For example, the abundance of bumble bees in the tropical agricultural highlands of Guatemala increased with the increase in forests and semi-natural vegetation in local areas, but was not influenced by season [181]. Habitat loss and climate change also affect honey bees worldwide, causing pollinator losses [182,183,184].

## 6. Bee Pollination vs. Non-Bee Pollination

Bees are considered the most effective pollinators; however, the contribution of other insect pollinators cannot be considered negligible, as they serve to increase and stabilize crop pollination and rely on these plants for the supply of pollen and nectar [17,149,184]. The main groups of other insect pollinators are butterflies, moths (Lepidoptera), some flies (Diptera), and beetles (Coleoptera) [17,185].

### 6.1. Hoverflies vs. Bees

Hoverflies (Diptera: Syrphidae) are considered the most anthophilous family in the order Diptera [186]. *Episyrphus balteatus* DeGeer is one of the most common hoverfly species to usually be found in agricultural areas, and several recent studies have confirmed its contribution as a pollinator of many crops around the world [187]. One crop that is pollinated by hoverflies *E. balteatus* is oilseed rape, which is an important crop in temperate regions. It has been shown that when *E. balteatus* is involved in the pollination services of this crop, sufficient numbers of seeds per pod are produced, demonstrating the ability of *E. balteatus* as a pollinator of edible crops [188]. The drone fly, *Eristalis tenax* L., has also been reported as a successful pollinator of numerous managed crops, such as pak choi (*Brassica rapa* subsp. *chinensis*) and onion in New Zealand [189], onion, spring turnip rape (*Brassica rapa* L. subsp. *oleifera*), and carrot (*Daucus carota* L. subsp. *sativus*) in Germany [190], sweet pepper (*C. annuum*) in Canada [191], and kiwifruit in Italy [192]. Consequently, according to Brad and Megan, *E. tenax* L. is often kept in large numbers in fields during the crop flowering period [189].

A study in Germany looked at the impact of pollination by the solitary mason bee *O. rufa* and two hoverfly species (*E. tenax* and *E. balteatus*) on oilseed rape. This study showed that the fruit yield and number of seeds per pod improved with an increase in bee abundance relative to hoverfly abundance, and that five-fold higher density of hoverflies than red mason bees were required to achieve the same fruit sets and yields. Thus, mason bees were more effective pollinators of this crop than hoverflies [122].

### 6.2. Butterflies vs. Bees

Around 180,000 species of butterfly and moth (Lepidoptera) are reported and make up to about 10% of all recognized insect species. Butterflies represent approximately 10% of Lepidoptera [193] and tend to visit psychrophilic flowers that offer small to medium volumes of dilute nectar. These flowers are characterized by brightly colored petals with a mild and pleasant aroma and a flat platform that enables the butterflies to land in the inflorescence. Large- to medium-sized butterflies act as pollinators via their wings [194]. *Gloriosa minor* Rendle (Colchicaceae) is a dry land floral plant in Kenya that depends on butterflies for pollination, and consequently the production of seeds, which are a source of colchicine [195]. *Caesalpinia pulcherrima* was pollinated mainly via butterflies which carry the pollen on their wings [196].

*Angadeniaberteroi* is an endangered species that has large, showy, yellow, and tubular flowers with no notable fragrance. The tubular shape of these flowers gives them a complex structure, so any pollinators that are attracted to this plant should have body parts that are specifically adapted to this morphology, including mouthparts that are long enough to find the nectar [197,198]. Both bees and butterflies visit *A. berteroi*, allowing a comparison of their efficiency. The long-tongued bees’ heads are wider than the apical portion of the pollen chamber, forcing them to touch the reproductive parts of the flower, and these bees rarely revisit the same flowers. Long-tongued bees appear to be efficient in gathering nectar and transporting pollen [117,199]. By contrast, the two groups of butterflies that visit the flowers [skippers (Hesperiidae and non-skippers) carry very small amounts of pollen on their proboscides, do not deposit this pollen on the stigmas of the flowers, and frequently visit the same flowers, thus appearing to act as nectar thieves. The frequent visitation of the same flower by an insect is known to have negative effects. For instance, flower re-visitation can lead to abortion of the fruit and ovule due to self-pollen deposition on the stigma, explaining why the mouthparts are associated with the efficacy of the pollination process [117].

### 6.3. Moths vs. Bees

Moths can be categorized as having a crepuscular or nocturnal lifestyle and are recognized as one of the main pollinators of a large variety of plant species in different habitats around the world [200]. The information on the role of moth pollination in natural habitats is available where about 227 flowers have been pollinated by moths [193]. Moths are frequent floral visitors, and there are a number of encounters between plant species and moths. Moths pollinate approximately 40% of plant species in rural landscape environments, such as meadows, pastures, old farms, field edges, and roadsides. Consequently, the role of moths in agricultural environments is often attributed to their pollination of non-crop plants, which contributes to increasing the biodiversity in agro-ecosystems, offering a widely appreciated ecological function [201,202]. Moths may also pollinate some unique plant species, such as some orchids. At present, however, the role of moths in pollination is likely underestimated due to the limited number of studies on this topic [203,204,205].

An evaluation of insect pollination levels on sunflower crops in the central Darling Downs during the day and night showed that Western honey bees were the most recurrent visitors, with populations averaging 65.3 bees per 100 flower heads across 42 crops through mid-morning [110]. By contrast, *Helicoverpa armigera* Hübner moths were observed visiting the plants during the night, averaging 3.9 individuals per 100 flower heads between 7 and 8 pm and being registered in 33 crops. Thus, moths visited the flowers for less than 2 h per night whereas bees were active for 9 h. The small population size and low level of activity of moths indicated that bees played a significant role in sunflower pollination in this area [110].

### 6.4. Beetles vs. Bees

Beetles (Coleoptera) belong to one of the most diverse insect orders and their role in pollination systems is increasingly being recognized. Some flowering plants depend on pollination by certain types of beetles. For example, species in the subfamily Cetoniinae (Scarabaeidae) are common pollinators in the tropics [206]. More than 184 species of angiosperms are exclusively pollinated by beetles (e.g., Magnolia in Magnoliaceae) [207,208]. Some beetles use flowers as rendezvous sites besides their usage in their food which enhances their role as pollinators. Unlike tropical pollinators, beetles depend on odor to find flowers, while Hopliine beetles exclusively use visual indications and, even without nourishment or smell, are attracted to bright colors [209]. Beetles are always associated with the pollination of open bowl-shaped flowers [207]. Beetles were found to be the second most important insect group contributing to pollination services in both Lambir (27%) and Kakachi (17%) in Malaysia and India, respectively. Both bees and beetles together represent more than 60% of the pollination services of tree species in Lambir and 34% of those in Kakachi [210]. Bees play the main pollinator role in Lambir (32%) followed by beetle-pollinated species (20%) [211], but the beetle *Hopliini* sp. (Scarabaeidae) is one of the most effective pollinators in the southwestern area of Cape Province and Namaqualand [212,213]. Therefore, there is a need for further studies to clarify the ecological role of beetles and their effectiveness as pollinators [214].

### 6.5. Thrips vs. Bees

Thrips (Thysanoptera) are pollinators of plant species, however they are still poorly studied [215]. These tiny insects have piercing-sucking mouthparts and are usually noticed on flowers, where they depend on nectar, pollen, or the cell content of plant tissues in their food [216]. Thrips have been noticed in the flowering period of coffee species *C. arabica* L. and *C. canephora* L. in the southern state of Chiapas, Mexico in three flowering seasons (2013–2015). Several species of thrips on coffee flower were noticed to be carrying a few pollen grains on their bodies [217]. Bees have increased fruit production of coffee by 50% more than wind in shaded coffee agro-forests, South India. The role of other insect visitors with bees including Thysanoptera in coffee was insignificant as they did not touch the flower anther or stigma enough times in addition to performing infrequent visitation [115].

### 6.6. Wasps vs. Bees

Social wasps (Hymenoptera) are among the pollinators in the Neotropical region. As predators, they can behave as flower visitors [218,219]. Many factors attract wasps to flowers such as flower color and shape. Wasps are attracted to reddish brown, dirty purple, and dirty brown flowers. Schremmer (1962) has noticed that wasps are attracted to small flowers with bulbous, wide entrances and sucrose-rich nectar [115,220]. Floral scent is one of the main factors attracting wasps. For example, social wasps are the main pollinators of *Epipactis helleborine* L. due to their scent [221,222]. Another study has been done on the coconut flower in the presence of wasps and honey bees. The wasps (*Polistes crinita* Felt) failed as a pollinator because of their disability in loading adequate amounts of pollen and their behavior in deterring the honey bee. In contrast, honey bees were effective as pollinators to coconut [95].

## 7. Conclusions

Bee pollination provides a wide variety of benefits to humanity, contributing to food processing, raw materials, medicines, fibers, social, cultural values, and the maintenance of biodiversity and environmental protections. Bees’ pollination has direct effects on the profitability and productivity of a substantial amount of global crop varieties, including most vegetables, seeds, and nuts, and some high-value agricultural products, such as coffee, cocoa, and rapeseed. Currently, 5–8% of all global crop production would be lost without the pollination services provided by bees, necessitating changes in the human diet and the expansion of agricultural lands to resolve shortfalls in crop production. Bees are faced with many challenges that can distort their lives, including shifts in land use, climate change, pesticides, genetics and cultivation management. Concerns regarding the decline of domestic and wild bees have intensified the need to encourage the usage of the wild pollinators on agricultural lands. As wild bee trips have increased with the development of high-diversity bee habitats in the surrounding landscape, the restoration of high-diversity bee habitats is necessary to increase free pollination levels. A secure atmosphere for bees should be provided to produce healthy crops. The use of insecticides and pesticides is damaging to human health because both crops and bee products become contaminated with agrochemicals that humans must eventually ingest. Although the roles played by non-bee pollinators cannot be ignored, bee pollination remains a precious asset that should be protected. Bee pollination must be enhanced not only to improve environmental balance but also to maintain food security worldwide. The role played by bees is important for worldwide crops and certain medicinal plants, with significant effects on quantity and quality. Researchers should focus their attention on studying the impacts that bees have on crop quality, which should provide more detailed data regarding how bees can alter the chemistry of certain crops.

## Data Availability

No new data were created or analyzed in this study. Data sharing is not applicable to this article.
